# ATP13A2 activates the pentose phosphate pathway to promote colorectal cancer growth though TFEB‐PGD axis

**DOI:** 10.1002/ctm2.1272

**Published:** 2023-05-26

**Authors:** Fan Zhang, Zhiwei Wu, Bowen Yu, Zhengping Ning, Zhixing Lu, Liang Li, Fei Long, Qionggui Hu, Chonglei Zhong, Yi Zhang, Changwei Lin

**Affiliations:** ^1^ Department of Gastrointestinal Surgery The Third Xiangya Hospital of Central South University Changsha China; ^2^ Department of Gastrointestinal, Hernia and Enterofistula Surgery People's Hospital of Guangxi Zhuang Autonomous Region Naning China; ^3^ Department of General Surgery Afliated Hospital of Xuzhou Medical University Xuzhou China; ^4^ Hunan Key Laboratory of Medical Genetics, School of Life Sciences Central South University Changsha China

**Keywords:** ATP13A2, pentose phosphate pathway, PGD, TFEB, tumour proliferation

## Abstract

**Background:**

The pentose phosphate pathway (PPP) is an important mechanism by which tumour cells resist stressful environments and maintain malignant proliferation. However, the mechanism by which the PPP regulates these processes in colorectal cancer (CRC) remains elusive.

**Methods:**

Closely related PPP genes were obtained from the TCGA and GEO databases. The effect of ATP13A2 on CRC cell proliferation was evaluated by performing in vitro assays. The connection between the PPP and ATP13A2 was explored by assessing proliferation and antioxidative stress. The molecular mechanism by which ATP13A2 regulates the PPP was investigated using chromatin immunoprecipitation and dual luciferase experiments. The clinical therapeutic potential of ATP13A2 was explored using patient‐derived xenograft (PDX), patient‐derived organoid (PDO) and AOM/DSS models.

**Findings:**

We identified ATP13A2 as a novel PPP‐related gene. ATP13A2 deficiency inhibited CRC growth and PPP activity, as manifested by a decrease in the levels of PPP products and an increase in reactive oxygen species levels, whereas ATP13A2 overexpression induced the opposite effect. Mechanistically, ATP13A2 regulated the PPP mainly by affecting phosphogluconate dehydrogenase (PGD) mRNA expression. Subsequent studies showed that ATP13A2 overexpression promoted TFEB nuclear localization by inhibiting the phosphorylation of TFEB, thereby enhancing the transcription of PGD and ultimately affecting the activity of the PPP. Finally, ATP13A2 knockdown inhibited CRC growth in PDO and PDX models. ATP13A2^−^/^−^ mice had a lower CRC growth capacity than ATP13A2^+/+^ in the AOM/DSS model.Our findings revealed that ATP13A2 overexpression‐driven dephosphorylation of TFEB promotes PPP activation by increasing PGD transcription, suggesting that ATP13A2 may serve as a potential target for CRC therapy.

## Graphical abstract

- ATP13A2 is upregulated in colorectal cancer (CRC) and associates with pentose phosphate pathway (PPP) activity.
- ATP13A2 promotes the transcription of PGD via inhibiting the phosphorylation of TFEB.
- Up‐regulation of PGD further activates the PPP and promote CRC growth.
- ATP13A2 is a potential therapeutic target for CRC.

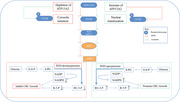

## 1 INTRODUCTION

Colorectal cancer (CRC) accounts for 10% of all cancer cases and 915 880 cancer‐related deaths in the past year, making it one of the most common cancers in the world.^1^ Uncontrolled and infinite proliferation is an essential *characteristic* of CRC.^2^ Effectively controlling the proliferation of tumour cells has always been the key to treatment. Tumour proliferation is a dynamic, complex and progressive process affected by many factors. For instance, tumours generally exhibit excessive pentose phosphate pathway (PPP) activation,^3^ enhanced autophagic flux^4^ and increased angiogenesis^5^ to resist oxidative stress and promote tumour growth. Multiple types of cancer exhibit protumourigenic activity through these pathways. It has been widely recognized that the PPP is a key component of promoting proliferation and resisting emergency environments, but the mechanisms regulating the PPP still require further research.

As an important branch of glycolysis, the PPP has been noted to play a significant role in the proliferation of CRC cells.^6^, ^7^, ^8^, ^9^, ^10^, ^11^ As the main pathway of glucose catabolism, the PPP is essential to cellular biological metabolism and antioxidant defenses.^12^ In addition to providing ribose‐5‐phosphate (R‐5‐P) for the de novo synthesis of RNA and DNA, PPP also provides the reducing equivalent NADPH for reductive biosynthesis.^13^ NADPH counteracts the excess reactive oxygen species (ROS) generated during rapid cell proliferation and promotes cell growth.^14^, ^15^, ^16^ Rapidly dividing cancer cells have evolved mechanisms to regulate the PPP and meet the requirements for cell survival.^17^ Therefore, identifying the regulatory genes of the PPP has become the key to tumour treatment. Some studies have shown that P53^18^ and Ras^19^ modulate PPP activity, providing novel targets for inhibiting the PPP. Although these genes were confirmed to regulate the PPP, the genes regulating the PPP remain unclear. In addition, the key catalytic enzymes of the PPP pathway, such as phosphogluconate dehydrogenase (PGD), have been proven to be up‐regulated in various tumours^20^, ^21^, ^22^ and play a crucial role in the PPP during tumourigenesis. However, the key role of the PPP mediated by PGD in the proliferation of CRC is poorly understood.

ATP13A2, a lysosome‐related transmembrane P5‐type ATP transportase,^23^ was first confirmed to play an important regulatory role in autophagy in Parkinson's disease.^24^, ^25^, ^26^, ^27^ Subsequently, it has also been shown to counteract oxidative stress^28^ and promote cell proliferation^29^ by regulating autophagy. In the literature related to ATP13A2 and cancer, only one study preliminarily confirmed that downregulation of ATP13A2 can suppress tumourigenesis by blocking autophagic flux in CRC.^30^ Since autophagy and PPP crosstalk occur in tumours,^31^ ATP13A2 likely exerts a similar effect on the PPP. However, the role of ATP13A2 in CRC has rarely been studied, and its relationship with the PPP is unknown.

In this study, we initially confirmed that ATP13A2 is a PPP‐related gene using bioinformatics analysis. Subsequently, we elucidated the molecular mechanism by which ATP13A2 activates the PPP by altering PGD. Finally, in vitro/in vivo analyses have confirmed that ATP13A2 was a potential diagnostic and therapeutic target for CRC. This study is the first to illustrate the role of ATP13A2 in activating the PPP in CRC and to refine the function of ATP13A2. The findings of our study provide a new direction for the clinical diagnosis and treatment of CRC in the future.

## 2 MATERIALS AND METHODS

### 2.1 CRC patients and samples

We obtained all our CRC tissues and adjacent normal tissues from the Third Xiangya Hospital of Central South University (Changsha, China). Tissue specimens are rapidly frozen within 10 min of isolation and stored at −80°C until use. The study was approved by Central South University's Medical Ethics Committee and was conducted with informed consent from patients. Here are the inclusion criteria for this study: (1) In the Third Xiangya Hospital of Central South University, the Department of Pathology found patients to have CRC after surgery; (2) radiotherapy or other treatments were not administered before surgery to the patients; (3) patients’ clinicopathological data, including sex, age, tumour size, tumour site, TNM stage and other information, were complete and correct.

### 2.2 Cell lines and cell culture

KeyGEN BioTECH (Nanjing, Jiangsu, China) provided SW480, SW620, RKO, LOVO and HT‐29 human CRC cell lines. The American Type Culture Collection (Manassas, VA, USA) provided a normal human intestinal epithelial cell line (FHC). A culture medium containing 10% fetal bovine serum (Gibco BRL, Gaithersburg, MD, USA) was used for all cells, which were incubated at 37°C, 95% humidity and 5% carbon dioxide.

### 2.3 An ATP13A2 cell line was generated using CRISPR/Cas9

According to a previous report,^32^ we generated a CRISPR/Cas9 knockout cell line. LentiCRISPR v2 (49535; Addgene) was subcloned with the sgRNA sequences targeting ATP13A2. Cotransfection of pCMV‐VSV‐G (8454; Addgene) and psPAX2 (12260; Addgene) packaging lentivirus plasmids with LentiCRISPR was performed in cells. Puromycin was used to eliminate noninfected cells after 48 h of replacing the medium with fresh medium containing puromycin. To generate a stable ATP13A2 knockout cell line, single clones of lentivirus‐infected cells were obtained and cultured in a 48‐well plate. Western blotting and Sanger sequencing technology were used to verify whether the gene was knocked out. All sequences are provided in Table S1.

### 2.4 Construction of plasmids, small interfering RNAs (siRNAs), short hairpin RNAs

In this study, Shanghai Genechem Co., Ltd. supplied the ATP13A2 overexpression and dual‐luciferase reporter gene plasmids. Three small interfering RNAs (siRNAs) targeting to PGD were designed and synthesized by GenePharma (Suzhou, China). Lentiviral short hairpin RNAs targeting ATP13A2 were obtained from Tsingke Biotech (Tsingke, China). A DNA sequence was performed on all plasmids used in this experiment.

### 2.5 Transfection

We transfected cells using Lipofectamine 3000 in accordance with the manufacturer's instructions. After transfection, the cells were maintained in complete medium at 37°C, 95% humidity, and 5% CO2 for 72 h. Transfection efficiency was confirmed by RT‒qPCR and Western blotting.

### 2.6 RNA extraction and quantitative reverse transcription‐PCR

In accordance with manufacturer's instructions, total RNAs were extracted from cells (cat# AG21024, Accurate Biology, China). Generated cDNA was prepared using the ReverTra Ace qPCR RT Master Mix (Yeasen, #11139ES10). We analyzed gene expression using a Roche LightCycler 480 using Vazyme's SYBR Green Master Mix (#Q711‐02). The primer sequences are shown in Table S1 for all primers from Tsingke Biotech (Tsingke, China).

### 2.7 Western blot assays

Using 1% PMSF (KeyGEN BioTECH) containing RIPA buffer (KeyGEN BioTECH), proteins were extracted from whole cells and tissues. The nucleus and cytoplasmic proteins are extracted from the cells according to the manufacturer's protocols (KeyGEN BioTECH, # KGA826). In brief, special reagents are added to the cells and centrifuged (1000 rcf), resulting in a final upper layer of cytoplasmic proteins and a lower layer of nuclear proteins. Using SDS‐PAGE, equal amounts of protein were separated and transferred to PVDF membranes (Millipore, CA, USA). After blocking with BSA, a protein‐loaded membrane was incubated with primary and secondary antibodies. Listed in Table S2 are the antibodies used in this study.

### 2.8 Cell proliferation and apoptosis assays

Cell proliferation was assayed with CCK‐8 (Yeasen, #BS350A) and the Click‐iT EdU Microplate Assay Kit (Yeasen, #BL915A), according to the manufacturer's protocols. In brief, medium containing CCK‐8 (10%) reagent was put into a 96‐well plate and subsequently incubated at 37°C and protected from light for 1 h. A 450‐nm wavelength was used to measure absorbance. The Annexin V‐FITC/PI Apoptosis Detection Kit (Yeasen, #BL110A) was used to detect the level of apoptosis. Cells were collected and washed twice with precooled PBS, followed by the addition of Annexin V‐FITC and PI Staining Solution, and reacted for 10−15 min at room temperature, protected from light. Cell apoptosis analysis was conducted by assessing stained cells on a fluorescence‐activated cell sorter (FACS).

### 2.9 ROS level analyses

The ROS Assay Kit (Biosharp, # BL714A) was used to detect the level of ROS. Specifically, the cells were treated with H2DCFDA, which was prepared by diluting it 1:1000 with serum‐free culture medium to achieve a final concentration of 10 μM. Following a 30‐min incubation at 37°C, the cells were washed twice with serum‐free culture medium. A FACS analysis was also conducted to determine the levels of intracellular ROS.

### 2.10 Glucose uptake and lactate assay

For analysis of glucose and lactate concentrations, glucose, and lactate assay kits (Biosharp, # BL863B, #BL868B) were employed in accordance with the manufacturer's recommended guidelines. Specifically, after adding reagents from the commercial kits and incubating the samples in a light‐protected environment at 37°C for 30 min, absorbance values were recorded at 450 and 520 nm. After subtracting the blank control values, the glucose values for the experimental samples were obtained, and lactic acid content was assessed according to the standard curve formula (*y* = 13.403x − .0119).

### 2.11 Determination of NADPH levels

The NADP^+^/NADPH Quantitation Kit (Biosharp, # BL860B) was used to measure NADPH levels, following the manufacturer's protocol for colorimetric analysis. Briefly, the cells were collected, the extract was added and ground in an ice bath, incubated at 60°C for 30 min, and centrifuged at 10,000 × g for 10 min at 4°C. After centrifugation, the supernatant was cooled on ice before subsequent measurement. The absorbance at 450 nm was measured. The NADPH values were obtained according to the standard curve formula (*y* = 8.6631x − .0013).

### 2.12 Immunofluorescence and microscopy

Cells were seeded into confocal dishes (Biosharp, #BS‐20‐GJM) and grown to 30%−50% confluence. Cells were first fixed with 4% paraformaldehyde, then permeabilized with .5% Triton X‐100 (v/v) for 30 min. After a blocking step with 3% BSA in PBS for 1 h, primary antibodies were added and the samples were incubated overnight.

After primary antibody incubation, the cells were treated with Alexa Fluor 488‐conjugated goat anti‐rabbit secondary antibodies (Abmart, #M213211) at a dilution of 1:400 for 1 h at room temperature. The cell nuclei were then stained with DAPI for 10 min. Finally, samples were imaged by confocal laser scanning microscopy (Zeiss, Oberkochen, Germany) and analyzed with ZEN Imaging Software 2.6 (blue edition).

### 2.13 Liquid chromatography‒mass spectrometry analysis of cell metabolites

[1,2‐^13^C]‐glucose and [U‐^13^C]‐glucose were purchased from Sigma (#453188, #389374). Glucose‐free DMEM (Sigma, #11966025) supplemented with 25 mM glucose was added to the cell culture dishes and incubated for 12 h. Approximately 1 × 10^7^ cells were then rinsed twice with cold PBS, and the cells were subsequently covered with liquid nitrogen to rapidly stop cell activity. After the liquid nitrogen evaporated, ice‐cold methanol was added, and all cells were covered with methanol and stored at −80°C for more than 30 min. Then, 100 μl of precooled ultrapure water was added, and the sample was mixed gently using a cell spatula. More than 90% of the cells were removed from the culture material into the methanolic water suspension, and the cells were collected along with the solvent. The samples were analyzed by Prof. Leader Shanghai. Specifically, the samples collected were processed by 5 cycles of 1 min of ultrasonication and 1 min intervals in an ice‐water bath and then allowed to stand for 30 min at −20°C. After centrifugation at 15000 rcf for 15 min at 4°C. The supernatant (1 mL) was dried and then reconstituted with 50 μL of 50% aqueous acetonitrile (1:1, v/v). Chromatographic separation was carried out on a ThermoFisher Ultimate 3000 UHPLC system with a Waters BEH Amide column. A 2 μL injection volume and a .35 mL/min flow rate were used. The mobile phase consisted of a mixture of water (phase A) with 15 mM ammonium acetate (pH = 8.5) and acetonitrile/water (90:10, v/v) (phase B). Linear gradient elution was performed with the following program: 0−2 min, 90% B; 14 min, 75% B; 15 min, 65% B; 15.2–16.9 min, 50% B; 17−20 min, 90% B. Finally, the eluents were analyzed in HESI‐ mode on a ThermoFisher Q Exactive Hybrid Quadrupole‐Orbitrap mass spectrometer (QE).

### 2.14 Dual‐luciferase reporter gene assay

The assay to measure the activity of dual luciferase reporter was carried out following the instructions provided in the manual for the Dual Luciferase Reporter Assay Kit (Vazyme, #DD1205‐01). Specifically, the cell culture plate was left at room temperature for 30 min to allow the plate temperature to equilibrate to room temperature. Subsequently, Duo‐Lite Luciferase assay reagent was added at an equal volume as the cell culture medium for the sample to be tested, and the sample was mixed well. After 10 min at room temperature, firefly luciferase luminescence was detected, and Duo‐Lite Stop and Lite assay reagent was added at an equal volume as the original cell culture medium for the sample to be tested, and the sample was mixed well. After an additional 10 min, the Renilla luciferase luminescence was measured.

### 2.15 Chromatin immunoprecipitation assay

The Pierce Agarose Chromatin immunoprecipitation (ChIP) Kit (Abcam, #ab500) was used to perform ChIP assays, following the manufacturer's protocol. ChIP DNA was purified, and subsequent qPCR experiments were conducted using SYBR Green Master Mix (Vazyme, #Q711‐02). A flank region with no signal was used as a negative control. The primer sequences are shown in Table S1.

### 2.16 Animal experiments

Male BALB/c athymic nude mice (4−6 weeks of age, 18−20 g) were provided by the Department of Laboratory Animals of Central South University (Changsha, Hunan, 260 China) and were randomly assigned to experimental groups. The mice were then injected subcutaneously with equal numbers of cells. Starting on day 5 or 7 after injection, tumour formation in the mice was observed every 3 days, and tumour volume and mouse weight were measured weekly. All animal studies were conducted with official approval from the Animal Research Ethics Committee.

### 2.17 CRC patient‐derived organoid model

According to the standard operating procedures (SOPs) for patient‐derived organoid (PDO) (https://pdmr.cancer.gov/sops) and previously published protocols,^33^ we successfully established PDO models from human colorectal adenocarcinoma samples. In addition, after constructing the PDO model, we performed STR analysis on both the parental CRC samples and our PDO model to confirm the consistency between our PDO model and the parental tissue source at the genomic level. Subsequently, the lentivirus was transfected into the PDO model as previously described. The growth state and size of the PDO were observed daily.

### 2.18 CRC patient‐derived xenograft model

In accordance with the SOPs for the patient‐derived xenograft (PDX) provided by the PDMR and previously published protocols,^34^ we successfully established PDX models from human colorectal adenocarcinoma samples. When the tumour size was 50–100 mm^3^, lentiviral particles (1 × 10^7^ TU) were injected into the left and right tumours of each mouse, respectively. Tumour volumes were calculated as previously describedand tumour diameters were then measured weekly.

### 2.19 AOM‐DSS animal model

The AOM‐DSS model was constructed with reference to Zhang et al.^35^ In brief, mice were intraperitoneally injected with AOM (10 mg/kg body weight) on day 0, followed by one cycle of DSS (3% w/v) dissolved in drinking water on day 3 for 3 days total; then the mice were provided with DSS‐free water for 7 days. This procedure was continued for five cycles, and the intestines of the mice were dissected to observe the tumour condition.

### 2.20 Statistical analysis

GraphPad Prism 9.0 and SPSS 26.0 were used for data processing. Each experiment has three independent measurements where data are presented as the mean + SD or mean + SEM. Two‐sided Student's *t* test was used to calculate *p* values. Statistical significance is indicated as *p ≤ .05.

## 3 RESULTS

### 3.1 Screening and verification of ATP13A2 as a novel PPP‐related gene

**FIGURE 1:**
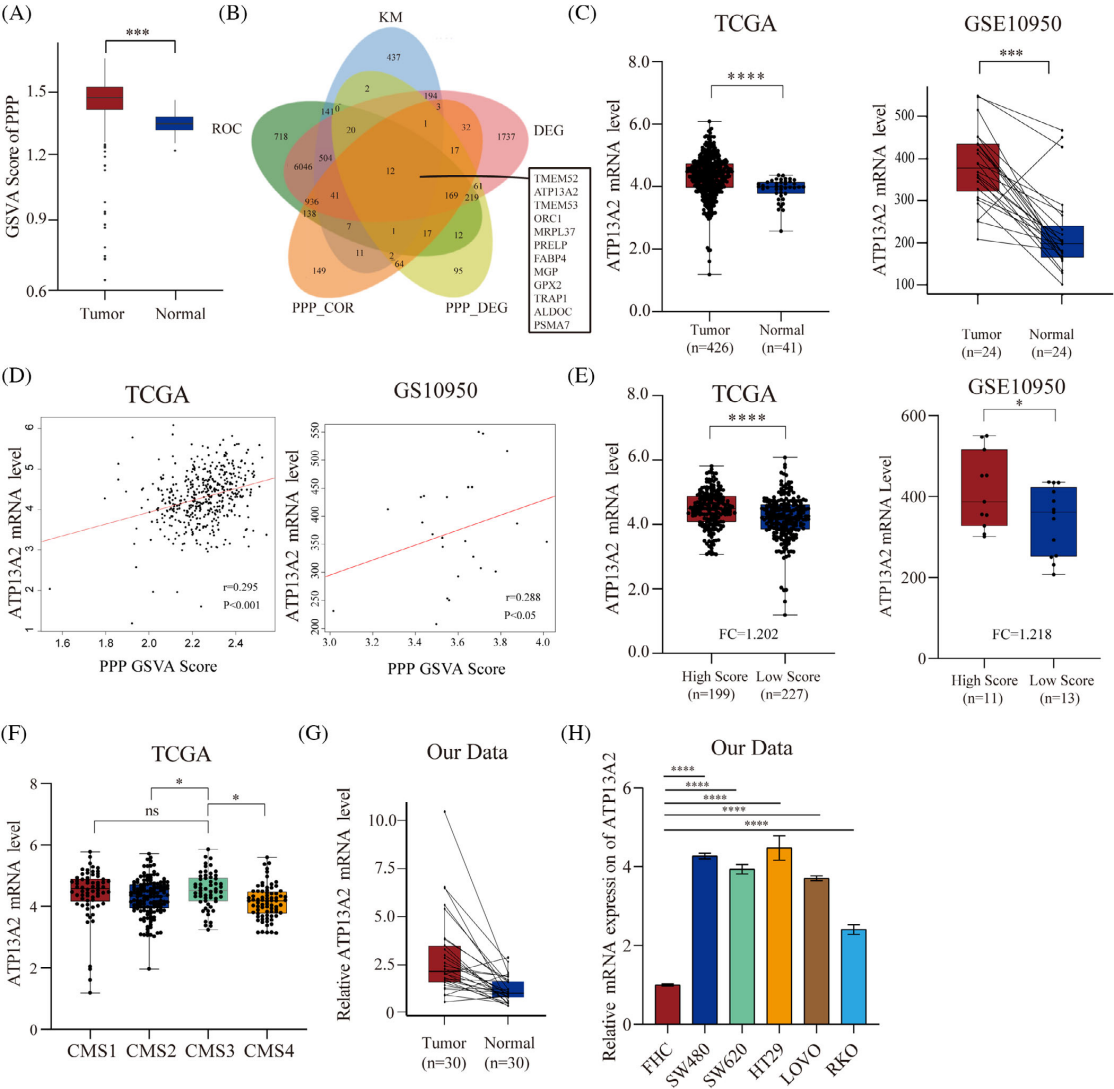
ATP13A2 is a novel pentose phosphate pathway (PPP)‐related gene and is expressed at high levels in colorectal cancer (CRC). (A) PPP activation in the tumour and normal tissues was determined by the GSVA enrichment score. (B) A common set of 12 genes was identified at the intersection of the 5 datasets. (C) ATP13A2 expression in CRC samples from TCGA and GEO databases. (D) Correlation between ATP13A2 expression and the GSVA enrichment score of the PPP. (E) Comparison of ATP13A2 expression in the high and low groups in terms of PPP GSVA scores. (F) ATP13A2 expression in different consensus molecular subtypes (CMSs). (G) ATP13A2 mRNA levels in 30 paired CRC tumour (T) and adjacent normal tissues (N) determined using quantitative reverse transcription‐PCR (qRT‒PCR). (H) ATP13A2 mRNA levels in FHC, SW480, SW620, HT29, LOVO and RKO cells determined using qRT‒PCR. All data are presented as means ± SD (*n* = 3 independent experiments). *p ≤ .05; **p ≤ .01; ***p ≤ .001; ****p ≤ .0001.

We obtained data from CRC and paracancerous tissues from TCGA database to screen novel PPP‐related genes involved in CRC. We first performed GSVA to determine whether a difference in the degree of PPP activation was observed between tumour and normal samples, and tumour group scores were higher than normal group scores (Figure 1A). Subsequently, we obtained 12 common genes from five gene sets: differentially expressed genes between tumour and normal tissues, genes with high diagnostic value, PPP‐related genes, differentially expressed genes between the high and low PPP activation groups, and prognostic genes (KM analysis) (Figure 1B). The GEO database (GSE10950) was used to validate the screened genes. Ultimately, only the ATP13A2 showed consistent trends across the two databases (Figure 1C–E).In addition, we detected higher ATP13A2 expression in the CMS3 type among molecular subtypes of CRC in TCGA (Figure 1F), and the CMS3 type of CRC is mainly characterized by the dysregulation of various metabolic pathways.^36^ Therefore, we selected ATP13A2 as our research object.

As a verification of the results of the analysis of these publicly available databases, we assessed the expression levels of ATP13A2 in 30 pairs of CRC tumour tissues and adjacent normal tissues. A significant increase in ATP13A2 expression was found in cancer tissues from patients with CRC compared with adjacent normal tissues (Figure 1G). In our next step, we summarized the clinicopathological characteristics of the 30 patients in the first cohort, and tumour sizes were larger in patients with high ATP13A2 expression than in those with low ATP13A2 expression (Table 1). Furthermore, we examined ATP13A2 expression levels in five human CRC cell lines (HT‐29, SW620, RKO, LOVO, and SW480) and one human normal colonic epithelial cell line (FHC), and ATP13A2 was expressed at high levels in the CRC cell lines (Figure 1H). Similar results were obtained from the CCLE database (Figure S1A). In addition, we also noticed that CRC cells with high levels of ATP13A2 expression had a relatively higher proliferation rate (Figure S1B). In previous studies of Parkinson's disease, ATP13A2 was always accompanied by a higher mutation rate; therefore, we subsequently explored the ATP13A2 mutation status in tumours. We used the cancer cell mutation database COSMIC (http://cancer.sanger.ac.uk/cosmic) to analyze ATP13A2 mutations. As shown in Figure S1C, ATP13A2 has a mutation frequency of only 4% in CRC, indicating that its mutation does not explain its role in tumours. Subsequently, using the data from TCGA database, we explored the DNA methylation of ATP13A2 in CRC, the result showed that ATP13A2 demonstrated DNA hypomethylation in CRC compared with normal samples. This may partly explain the reason of ATP13A2 up‐regulation in CRC (Figure S1D). Taken together, these findings indicate that ATP13A2 is expressed at high levels in CRC and is tightly linked to the PPP.

**TABLE 1:** Clinic‐pathological characteristics of enrolled patients.

Clinical parameters		ATP13A2		
	Total	High (%)	Low (%)	*p*‐Value
Gender				
Female	21	9 (42.9)	12 (57.1)	.522
Male	9	5 (55.6)	4 (44.4)	
Age (years)				
≤60	4	2 (50.0)	2 (50.0)	.884
> 60	26	12 (46.2)	14 (53.8)	
Pathologic stage				
Stage I‐II	19	10 (52.6)	9 (47.4)	.389
Stage III‐IV	11	4 (36.4)	7 (63.6)	
Pathology T stage				
T1‐T2	8	4 (50.0)	4 (50.0)	.824
T3‐T4	22	10 (45.5)	12 (54.5)	
Pathology N stage				
N0	19	10 (52.6)	9 (47.4)	.546
N1	7	2 (28.6)	5 (71.4)	
N2	4	2 (50.0)	2 (50.0)	
Pathology M stage				
M0	29	14 (44.8)	15 (55.2)	.341
M1	1	0 (0)	1 (100.0)	
Tumour Size				
≥5 cm	11	9 (81.8)	2 (18.2)	.003
<5 cm	19	5 (26.3)	14 (73.7)	
Tumour Site				
Left colon	1	0 (0)	1 (100.0)	.628
Right colon	12	6 (50.0)	6 (50.0)	
Rectum	17	8 (47.1)	9 (52.9)	

### 3.2 In vitro and in vivo, ATP13A2 promotes CRC cell proliferation

Activation of the PPP plays an important role in tumour proliferation,^37^ and ATP13A2 may have the same role as a PPP‐related gene. Therefore, we first explored the effect of ATP13A2 on CRC growth.

**FIGURE 2:**
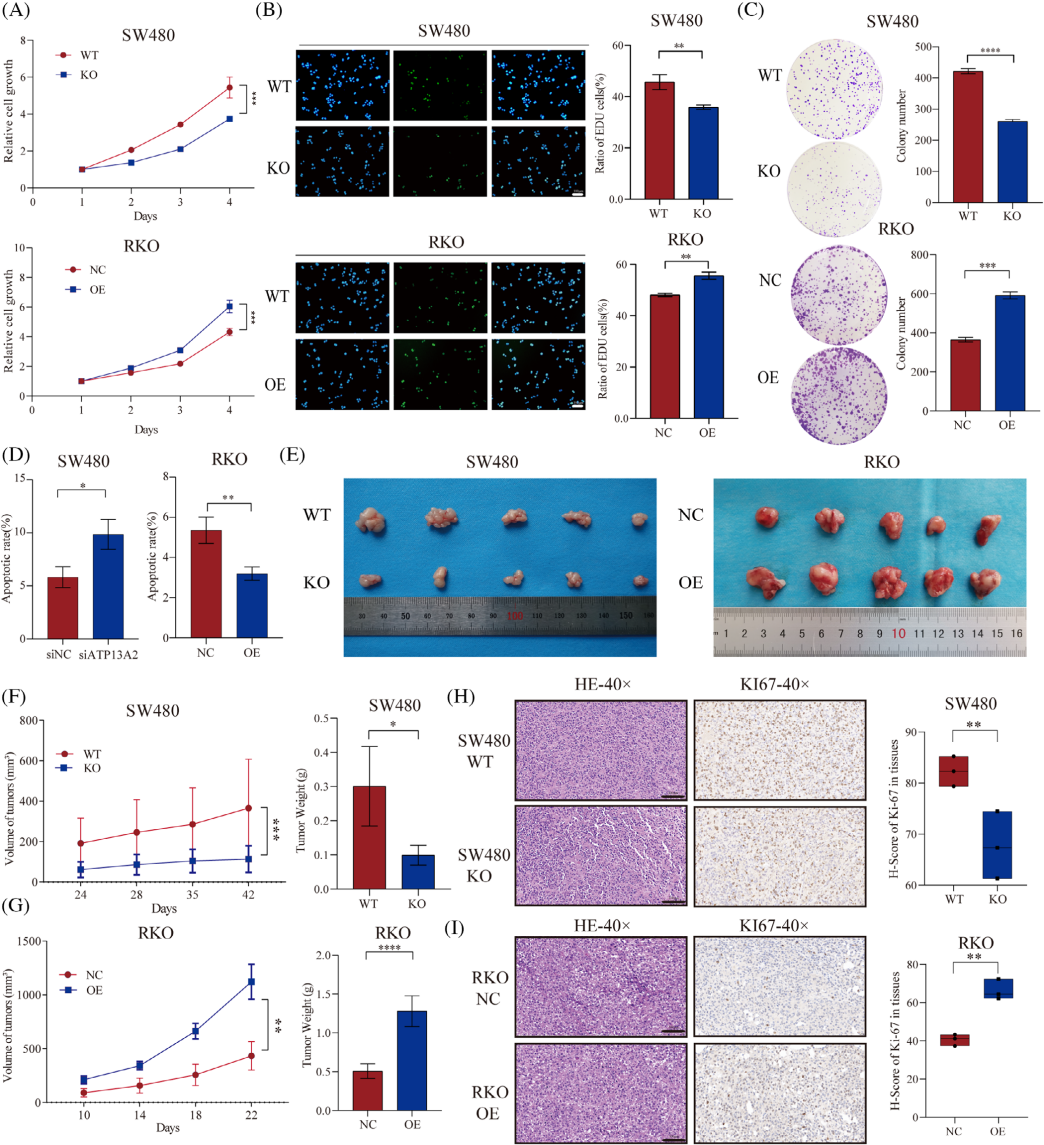
ATP13A2 promotes colorectal cancer (CRC) cell proliferation in vitro and in vivo. (A) Cell proliferation was assessed using a CCK‐8 assay. (B) EdU%, which refers to the EdU labelling index (%), was calculated as the number of EdU‐positive cells/total number of DAPI‐positive cells. (C) Self‐renewal ability was detected by performing plate colony formation assays. (D) Apoptosis rate (%) of each group. (E) Photograph and quantification of the size of excised subcutaneous tumours (*n* = 5 mice per group). (F and G) Tumour volume curve and tumour weight graph. (H and I) HE staining and Ki67 immunostaining of transplanted tumour tissues and Ki67 protein immunohistochemical histological score (H‐score). All data are presented as means ± SD (*n* = 3 independent experiments). *p ≤ .05; **p ≤ .01; ***p ≤ .001; ****p ≤ .0001.

We first constructed ATP13A2 knockout (ATP13A2‐KO) HT29 and SW480 cell lines, as these parental cell lines exhibit relatively high ATP13A2 expression (Figure S2A). Subsequently, RKO cell lines with relatively low expression were selected to construct ATP13A2‐overexpressing (ATP13A2‐OE) cell lines, as described in detail in Figure S2B. CCK‐8, EdU and plate colony formation assays revealed that ATP13A2 knockout inhibited the proliferation of CRC cells, while its overexpression produced the opposite results (Figure 2A–C, Figure S2C–E). Overexpression of ATP13A2 in ATP13A2‐KO cells can restore the effect induced by ATP13A2 knockout (Figure S2H,I). Flow cytometry indicated that knockout of ATP13A2 increased the number of apoptotic CRC cells, while its overexpression reduced the number of apoptotic CRC cells (Figure 2D, Figure S2F,G). Western blotting indicated that the knockout of ATP13A2 increased the levels of cleaved‐caspase3 and cleaved‐caspase7 while decreased the level of BCL‐2. Meanwhile, the overexpression of ATP13A2 showed the opposite trend. Therefore, these results confirmed that ATP13A2 could inhibit apoptosis in CRC cells (Figure S2J). Subsequently, we investigated the effect of ATP13A2 on CRC xenograft growth in vivo (Figure S2K). Notably, tumours derived from ATP13A2‐KO cells were smaller and lighter, whereas tumours derived from ATP13A2‐OE cells were larger and heavier than those in the control group (Figure 2E–G, Figure S2L–N). Ki67 expression was decreased in tumours derived from ATP13A2‐KO cells and increased in tumours derived from ATP13A2‐OE cells (Figure 2H,I, Figure S2O). More importantly, we also tested those apoptotic‐ related markers using samples from CRC xenograft. The results also showed that the ATP13A2 could regulate apoptosis in vivo (Figure S2P–R).

Based on these data, ATP13A2 promotes the growth of CRC both in vitro and in vivo.

### 3.3 ATP13A2 enhances the PPP

**FIGURE 3:**
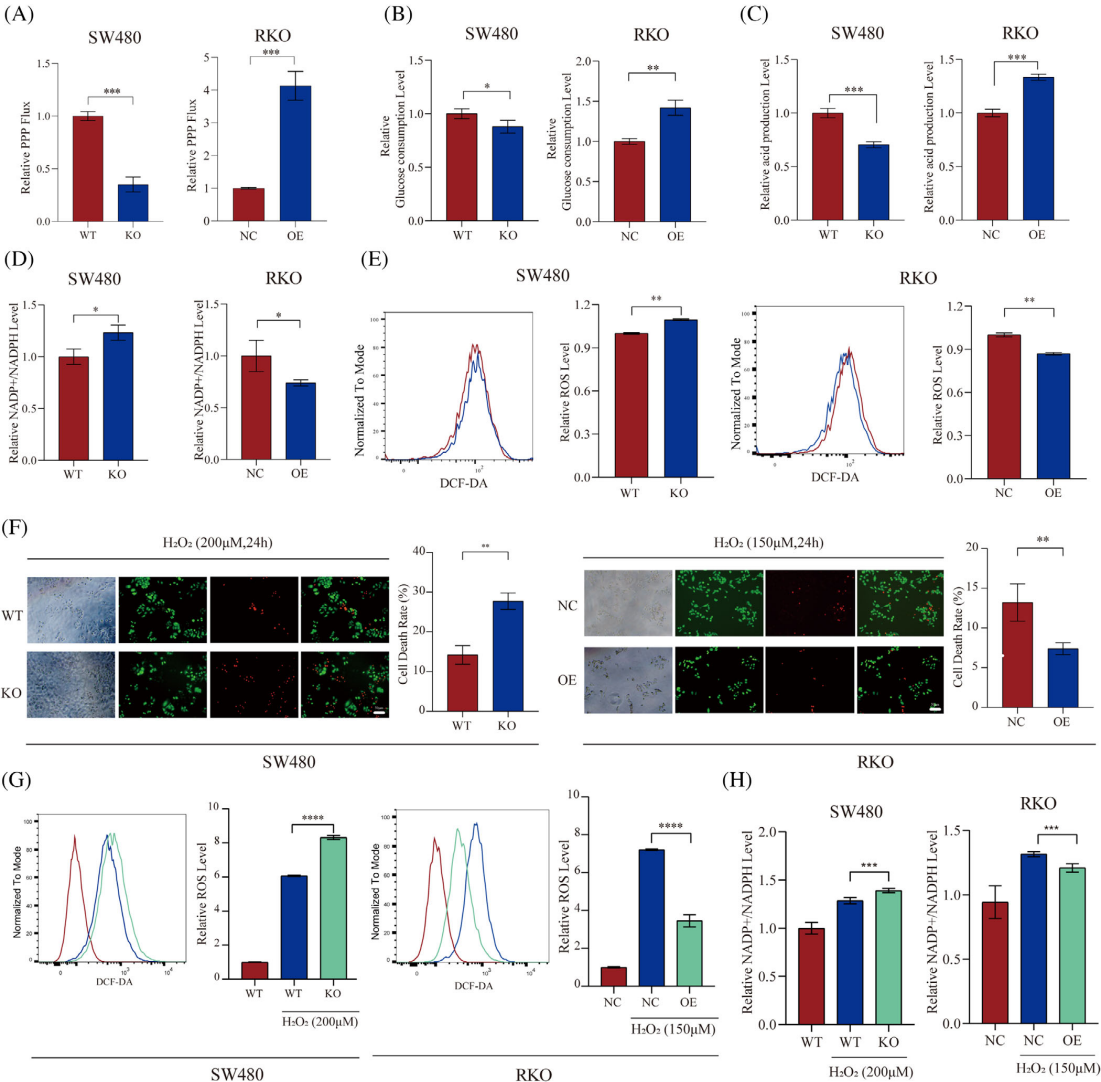
ATP13A2 enhances pentose phosphate pathway (PPP) activity in colorectal cancer (CRC) cells. (A) ATP13A2‐KO and ATP13A2‐OE cells were cultured for 12 h with complete medium (medium) containing 10 mM ^13^C‐1,2‐D‐glucose. The medium was used to analyze PPP flux with NMR. (B and C) Analysis of lactate and glucose uptake by ATP13A2‐KO and ATP13A2‐OE cell lines using the colorimetric method. (D) NADP^+^/NADPH levels in ATP13A2‐KO and ATP13A2‐OE cells. (E) Fluorescence‐activated cell sorter (FACS) analysis (left panel) and statistical results (right panel) for ROS levels in ATP13A2‐KO and ATP13A2‐OE cells. (F) Calcein‐AM/PI double staining of living cells and dead cells. Living cells were stained with Calcein‐AM (green), and dead cells were stained with PI (red). (G) FACS analysis (left panel) and statistical results (right panel) for ROS levels after treatment with 200 or 150 μmol for 24 h. (H) NADP^+^/NADPH levels in ATP13A2‐KO and ATP13A2‐OE cells after treatment with 200 μmol or 150 μmol for 24 h. All data are presented as means ± SD (*n* = 3 independent experiments). *p ≤ .05; **p ≤ .01; ***p ≤ .001; ****p ≤ .0001.

We first measured PPP flux to investigate the relationship between ATP13A2 and the PPP. [1,2‐^13^C]‐labelled glucose distinguishes between lactate produced via the PPP and that derived from the general glycolysis pathway,^38^ and the PPP flux was obtained by calculating the ratio. A reduction of 50%−60% in PPP flux is observed with ATP13A2 deficiency, while a significant increase is observed with ATP13A2 overexpression (Figure 3A, Figure S3A). Based on this observation, we speculate that the deletion of ATP13A2 may reduce glucose uptake. As expected, ATP13A2‐KO cells utilized less glucose and produced less lactate, whereas ATP13A2‐OE cells exhibited the opposite changes (Figure 3B,C, Figure S3B,C).

As the main pathway involved in NADPH production, the PPP plays a very important role in resisting oxidative stress and ROS.^39^ Therefore, we assessed NADPH and ROS levels in cells with different ATP13A2 expression levels to further explore the effect of ATP13A2 on the PPP. As shown in Figure 3D,E and Figure S3D,E, NADPH production decreased and ROS levels increased in ATP13A2‐KO cells compared to control cells, while ATP13A2‐OE cells showed the opposite trend.

Studies have shown that when cells are exposed to oxidants, they activate the PPP through a variety of pathways to combat oxidative stress.^39^ Researchers typically use H_2_O_2_ to mimic this state of oxidative stress.^40^, ^41^ Therefore, we added H_2_O_2_ to the cells to explore the effect of ATP13A2 on the highly activated PPP. We first added different concentrations of H_2_O_2_ to the cell culture medium, incubated the cells for 24 h, and then determined the number of viable cells to determine the optimal H_2_O_2_ concentration. The number of cells began to change when the H_2_O_2_ concentration of HT29 and SW480 cells was .2 mmol/L and that of RKO cells was .15 mmol/L (Figure S3F). We next used calcein‐AM/PI reagent to determine the number of viable cells 24 h after H_2_O_2_ was added. ATP13A2‐KO cells were more sensitive to oxidative stress and exhibited an increased cell death rate, while ATP13A2 overexpression was conducive to a better environment for cells to resist oxidative stress (Figure 3F, Figure S3G). Moreover, ATP13A2 knockout significantly increased ROS accumulation induced by H_2_O_2_ in cells compared with the control group, while ATP13A2‐OE cells showed the opposite trend (Figure 3G, Figure S3H). These results suggest that high ATP13A2 expression is beneficial for protecting the environment of CRC cells against oxidative stress. A subsequent analysis of NADPH production also generated similar results (Figure 3H, Figure S3I).

In general, ATP13A2 may be an important regulator of PPP activation. Therefore, the mechanism by which ATP13A2 activates the PPP is worthy of further exploration.

### 3.4 ATP13A2 enhances the PPP by regulating PGD expression

**FIGURE 4:**
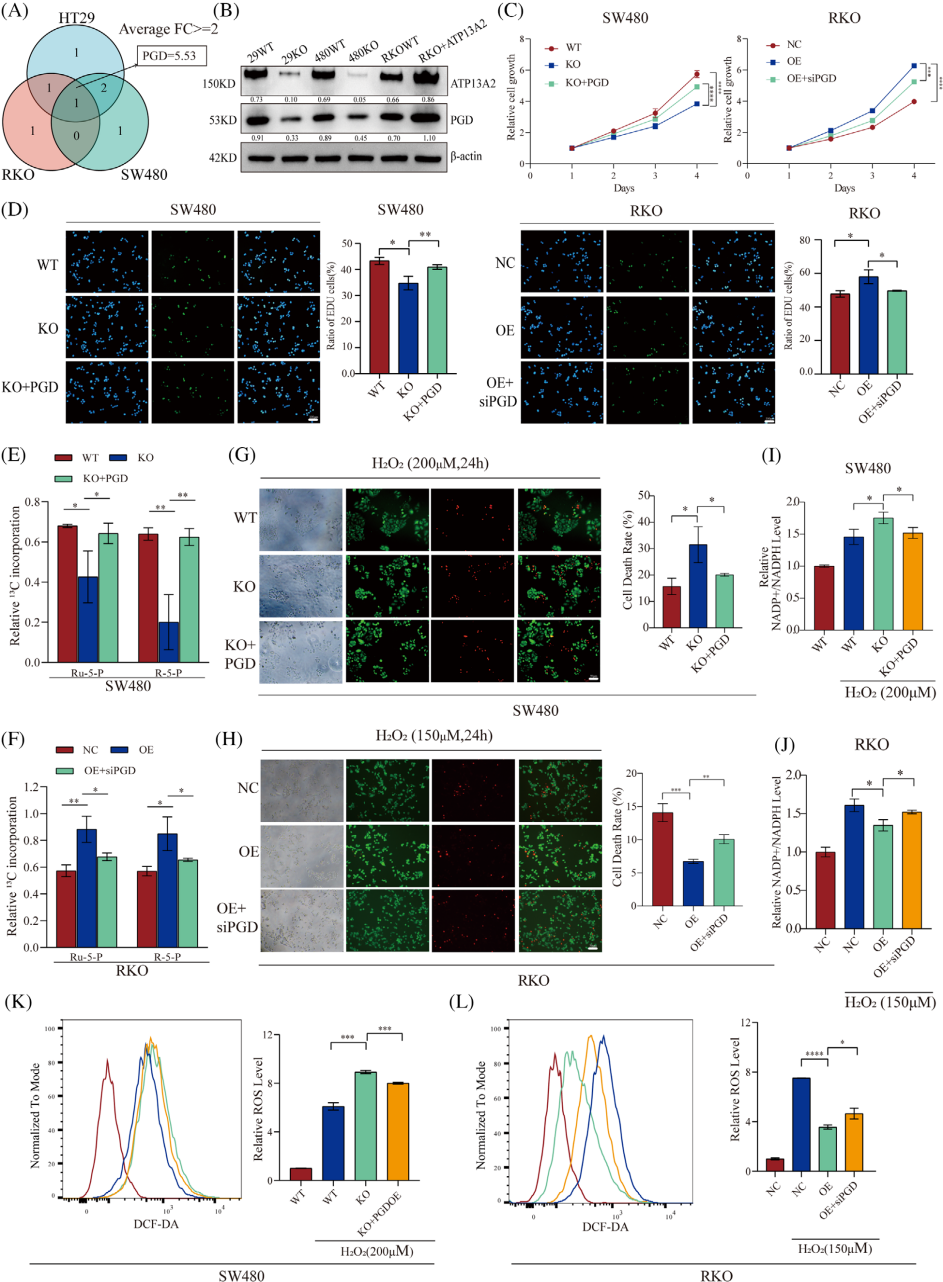
ATP13A2 enhances the pentose phosphate pathway (PPP) by regulating phosphogluconate dehydrogenase (PGD) expression. (A) PGD was identified at the intersection of the three datasets. (B) The expression levels of ATP13A2 and PGD in control and ATP13A2‐KO or ATP13A2‐OE cells were examined using western blotting. (C) Cell proliferation was assessed using a CCK‐8 assay. (D) EdU%, which is the EdU labelling index (%), was calculated as the number of EdU‐positive cells/total number of DAPI‐positive cells. (E and F) Using [U^13^C]‐labelled glucose, the levels of RU‐5‐P and R‐5‐P generated from PPP flux were detected using LC‒MS. (G and H) Calcein‐AM/PI double staining of living cells and dead cells. Living cells were stained with Calcein‐AM (green), and dead cells were stained with PI (red). (I and J) Fluorescence‐activated cell sorter (FACS) analysis (left panel) and statistical results (right panel) for ROS levels after treatment with 200 or 150 μmol for 24 h. (K and L) FACS analysis (left panel) and statistical results (right panel) for ROS levels after treatment with 200 or 150 μmol for 24 h. All data are presented as means ± SD (*n* = 3 independent experiments). *p ≤ .05; **p ≤ .01; ***p ≤ .001; ****p ≤ .0001.

Then, we detected the gene expression levels of PPP enzymes in ATP13A2‐KO and ATP13A2‐OE cells using quantitative reverse transcription‐PCR (qRT‒PCR) (Figure S4A) and constructed Venn diagrams to obtain the intersecting genes. PGD was a significantly altered gene in three datasets (Figure 4A), and the results were further verified by western blotting (Figure 4B). The CCK‐8 and EdU assays revealed that PGD overexpression reversed the inhibitory effect on ATP13A2‐KO cell proliferation, while PGD siRNA reduced the proliferation of ATP13A2‐OE cells (Figure 4C,D, Figure S4B,C).

PGD is the key enzyme in the PPP and mainly catalyzes the production of ribulose‐5‐phosphate (RU‐5‐P) and NADPH.^17^ RU‐5‐P also provides raw materials for the generation of ribose 5‐phosphate (R‐5‐P), which is a substance necessary for cell proliferation.^38^ We performed metabolic tracing of [U‐^13^C]‐labelled glucose in CRC cells using liquid chromatography‒mass spectrometry (LC‒MS) to confirm the effect of ATP13A2 on PPP flux through PGD. PGD reversed the decreases in the levels of key PPP products (RU‐5‐P and R‐5‐P) caused by ATP13A2 deletion (Figure 4E, Figure S4D). Transfection of the PGD siRNA into ATP13A2‐OE cells reversed the increased levels of key PPP products (RU‐5‐P and R‐5‐P) (Figure 4F), which further supports the hypothesis that ATP13A2 affects the PPP through PGD. Next, we exposed the cells to the oxidant H_2_O_2_. PGD clearly reversed the increase in the sensitivity of cells to oxidative stress caused by ATP13A2 knockout, as manifested by an increased number of surviving cells, increased NADPH production, and decreased ROS levels (Figure 4G,I,K, Figure S4E–G). After PGD was inhibited, ATP13A2‐OE cells showed increased sensitivity to oxidants (Figure 4H,J,L).

Collectively, our results suggest that ATP13A2 affects PGD expression to regulate the PPP, which not only promotes the proliferation of CRC cells but also enhances their ability to resist oxidative stress, supporting CRC cell survival in the real tumour environment.

### 3.5 ATP13A2 regulates PGD transcription via TFEB

**FIGURE 5:**
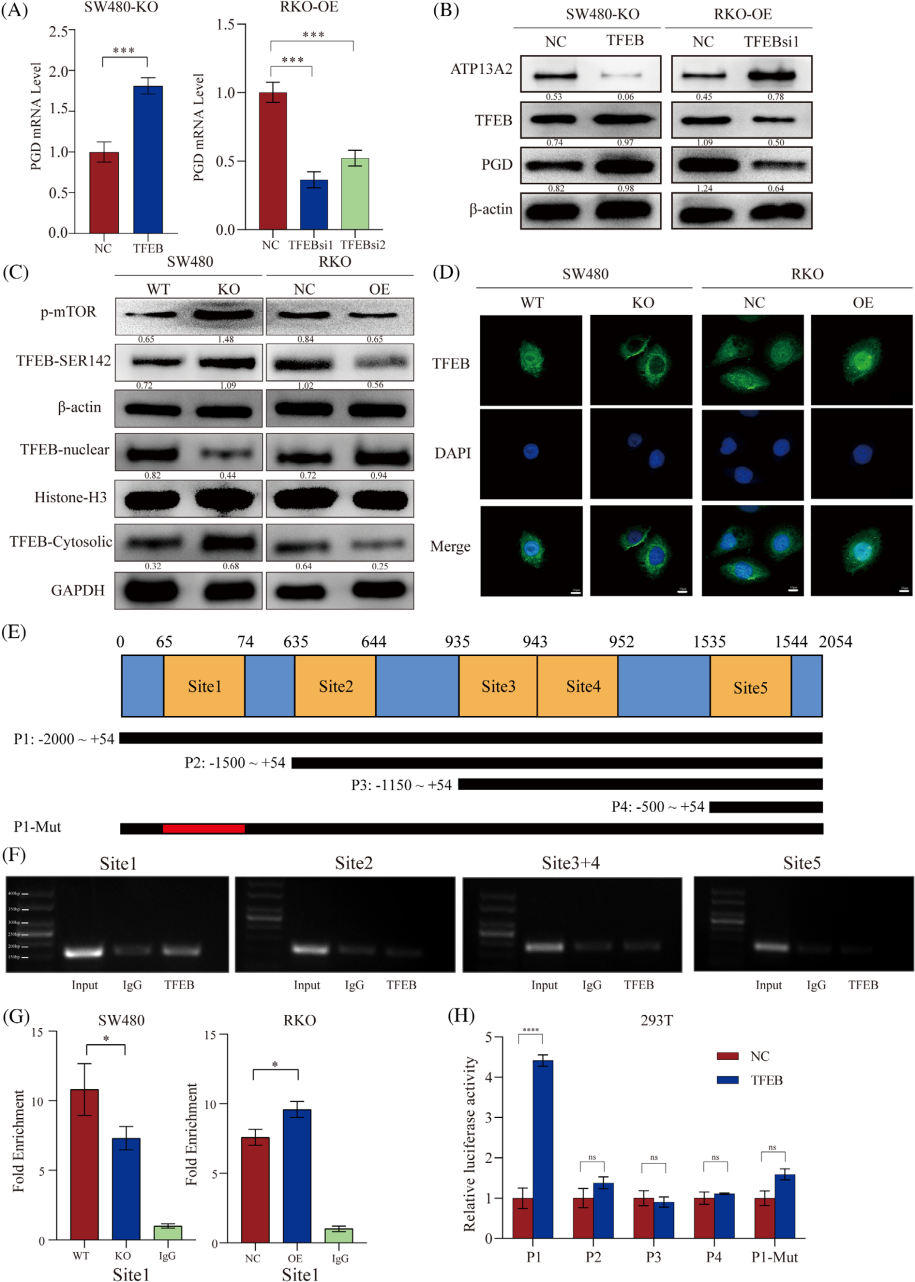
ATP13A2 induces phosphogluconate dehydrogenase (PGD) expression by increasing the nuclear translocation of TFEB and inhibiting its phosphorylation. (A) PGD expression in response to TFEB overexpression or TFEB knockout in ATP13A2‐KO or ATP13A2‐OE cells, as determined using quantitative reverse transcription‐PCR (qRT‒PCR) and (B) western blotting. (C) The phosphorylation levels of mTORC and TFEB in ATP13A2‐KO or ATP13A2‐OE cells and nucleoplasmic distribution of TFEB were detected using western blotting. (D) TFEB translocation to the nucleus was assessed by determining DAPI colocalization. ATP13A2‐KO cells or ATP13A2‐OE cells (and respective controls) were imaged using a confocal microscope (scale bar, 10 μm). (E) Schematic diagram showing the five potential TFEB binding sites and luciferase plasmids containing the promoter region of the PGD gene. (F) A ChIP analysis was performed in 293T cells. TFEB binding to the PGD promoter is presented. (G) ATP13A2‐KO and ATP13A2‐OE cells were used to perform ChIP. The primers designed to target the putative TFEB‐binding site 1 on the PGD promoter were used for qRT‒PCR. (H) A luciferase reporter assay was performed to evaluate interactions between TFEB and the target sites in the PGD promoter. All data are presented as means ± SD (*n* = 3 independent experiments). *p ≤ .05; **p ≤ .01; ***p ≤ .001; ****p ≤ .0001.

Our previous results showed that ATP13A2 affects the transcription of PGD, while previous studies have shown that ATP13A2 modulates TFEB activity to regulate downstream gene expression.^32^ Therefore, we speculate that ATP13A2 may regulate PGD transcription through TFEB. We overexpressed TFEB in ATP13A2‐KO cells and observed changes in PGD expression to further determine the role of TFEB in the effects of ATP13A2 and PGD. The trend for the change in the expression of the two molecules was consistent (Figure 5A,B), and the subsequent transfection of TFEB siRNA into ATP13A2‐OE cells yielded similar results. Accordingly, we speculate that ATP13A2 regulates PGD expression by TFEB.

Studies have shown that TFEB translocate from the nucleus to the cytoplasm when phosphorylated.^42^ And considering previous study have demonstrated that ATP13A2 suppresses TFEB via inhibition of mTORC1. We performed western blotting to clarify the effect of ATP13A2 on mTOR phosphorylation (activated mTORC1), TFEB phosphorylation and the process of entering and leaving the nucleus. The loss of ATP13A2 resulted in increased phosphorylation of TFEB‐S142, and more TFEB remained in the cytoplasm, while the up‐regulation of ATP13A2 produced the opposite result (Figure 5C, Figure S5A,B). Subsequently, we analyzed the effect of changes in ATP13A2 expression levels on the distribution of TFEB in the nucleus and cytoplasm using immunofluorescence staining and obtained similar results to those described above (Figure 5D).

We analyzed 0−2000 bp upstream regions of the PGD transcription start site to further explore the connection between TFEB and PGD. In the 2000‐bp promoter region of PGD, the putative TFEB binding sites were identified using Jaspar (http://jaspar.genereg.net).We identified five predicted binding sites for TFEB on the PGD promoter (Figure 5E). We verified the predicted sites using ChIP‐PCR experiments, and the results showed that only site 1 (+65–+74) is the likely binding site of TFEB in the PGD promoter region (Figure 5F). We then conducted ChIP‒qPCR to further compare the effect of altered ATP13A2 expression levels on the ability of site 1 to bind TFEB, and the results showed that down‐regulation of ATP13A2 expression impaired site 1 binding, while ATP13A2 overexpression exerted the opposite effect (Figure 5G). Next, we performed a dual luciferase experiment to verify the accuracy of the ChIP experiment. As shown in Figure 5H, TFEB effectively interacted with the promoter sequence only when the promoter sequence contained site 1. Regardless of whether this site was deleted or mutated, TFEB did not interact with the promoter region normally. To further understand the relationship between ATP13A2, TFEB and PGD, we tested the expression levels of TFEB phosphorylation and PGD in clinical samples of CRC patients by Western Blotting. The results showed that compared with adjacent normal tissues, phosphorylation of TFEB‐S142 decreased and PGD levels increased in CRC patients (Figure S5C). This result further indicated the dependency of ATP13A2/TFEB/PGD axis in CRC.

Based on these results, ATP13A2 may alter the expression of the PGD mRNA by regulating the TFEB phosphorylation level.

### 3.6 Inhibition of ATP13A2 reduces CRC growth in a clinical model

**FIGURE 6:**
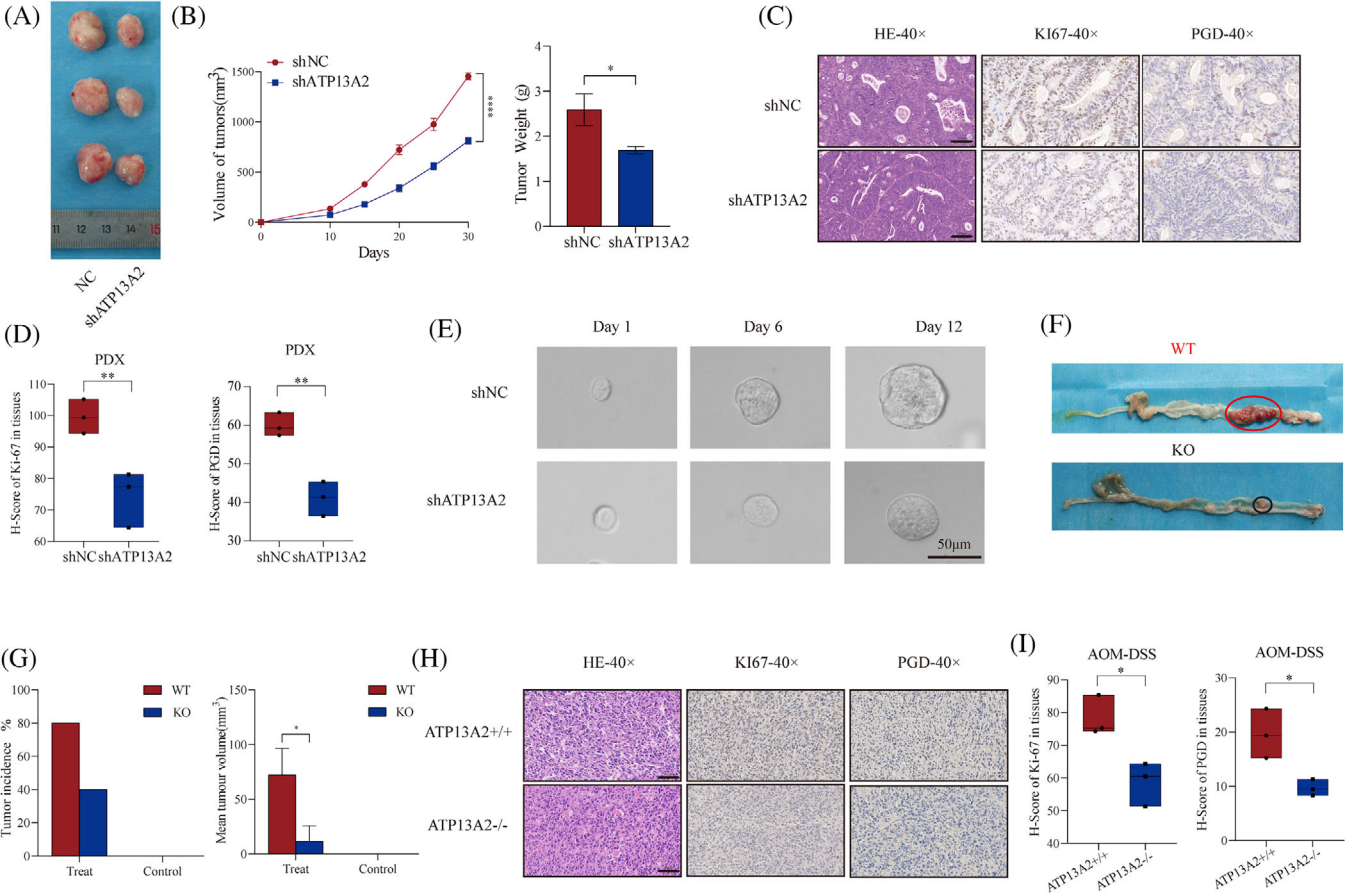
Inhibition of ATP13A2 suppresses colorectal cancer (CRC) growth in PDO, patient‐derived xenograft (PDX) and AOM/DSS models. (A) Images of tumours from mice in each group (*n* = 3 mice/group). (B) Tumour weights of CRC PDXs in each group. All data are presented as the means ± SD of *n* = 3 independent experiments. *p < .05; **p < .01; ***p < .001; ***p < .0001. (C) HE staining, Ki67 and phosphogluconate dehydrogenase (PGD) immunostaining in CRC PDXs. (D) The immunohistochemical histological score (H‐score) of Ki67 and PGD proteins. (E) The growth of CRC PDOs after transfection with the shNC or shATP13A2 lentiviral vector. (F) Representative macroscopic views of the tumour area in the colon after tumour initiation. (G) Calculation of the tumour incidence and weight between the ATP13A2^−/−^ and ATP13A2^+/+^ groups. (H) HE staining, Ki67andPGD immunostaining of the ATP13A2^−/−^ and ATP13A2^+/+^ groups. (I) The immunohistochemical histological score (H‐score) of Ki67 and PGD proteins. All data are presented as means ± SD (*n* = 3 independent experiments). *p ≤ .05; **p ≤ .01; ***p ≤ .001; ****p ≤ .0001.

We next established CRC PDO and xenograft (PDX) models to confirm the treatment effect of targeting ATP13A2 and to further investigate the potential clinical implications of ATP13A2 in CRC treatment. Then, we knocked down ATP13A2 with a lentivirus through intratumoural injection and found that the tumour volume and weight in the sh‐ATP13A2 group were lower than those in the control group (Figure 6A,B, Figure S6A,B). Furthermore, Immunohistochemical (IHC) staining showed that the levels of Ki67 and PGD protein were also decreased in the ATP13A2 knockdown plasmid‐treated group compared to the control group (Figure 6C,D).Similarity, the mRNA level of PGD also decreased in the ATP13A2 knockdown plasmid‐treated group compared to the control group (Figure S6C). Then, we knocked down ATP13A2 in PDOs with lentiviral vectors and found that ATP13A2 knockdown inhibited the growth of PDOs (Figure 6E). Finally, we constructed an AOM/DSS model to further evaluate the effect of tumour therapy targeting the ATP13A2 gene (Figure 6F, Figure S6D–F). ATP13A2^−/−^ mice also had a significantly lower number of tumours and smaller tumour size than ATP13A2^+/+^ mice (Figure 6G). IHC staining of mouse tumours revealed that ATP13A2^−/−^ mice displayed significantly lower Ki67 and PGD protein expression than ATP13A2^+/+^ mice (Figure 6H,I). As expected, the mRNA level of PGD in tumour of ATP13A2^−/−^ mice decreased (Figure S6G). In summary, targeting ATP13A2 exerted a good therapeutic effect on different CRC models.

In summary, this study illustrates the mechanism by which ATP13A2 overexpression promotes TFEB dephosphorylation and nuclear translocation. Then, dephosphorylated TFEB binds to the PGD promoter and promotes PGD transcription. Finally, the upregulated PGD enhances PPP activity and promotes CRC growth.

## 4 DISCUSSION

Cancer manifests metabolic reprogramming as a core characteristic. The PPP in cancer cells serves a dual function: it produces pentose phosphates needed for high rates of nucleic acid synthesis and generates NADPH necessary for cell survival during stressful conditions.^43^ Thus, the identification of therapeutic targets that inhibit the PPP has become the key to treating tumours. Despite the fact that the PPP has been targeted for cancer treatment,^44^ efficient anti‐PPP agents are not available in the clinic. Therefore, an attempt to identify more key genes regulating the PPP will undoubtedly be of great importance to find therapeutic targets that inhibit the PPP. In this study, we first identified ATP13A2 as a new gene regulating the PPP in TCGA and GEO databases and proved that ATP13A2 regulates the growth of CRC in vitro and in vivo. Then, we showed that overexpression of ATP13A2 enhanced the activity of the PPP by upregulating PGD expression. Furthermore, ATP13A2 overexpression caused TFEB dephosphorylation and nuclear translocation, which subsequently bound to the PGD promoter and promoted PGD transcription. Finally, our PDX, PDO and AOM/DSS models support the potential of ATP13A2 in clinical therapy.

For a long time, an autophagy disorder has been perceived as the main pathological feature of the dysregulation of ATP13A2, a lysosome‐related transmembrane P5‐type ATP transportase, and research on CRC and ATP13A2 is limited to the field of autophagy.^30^ However, autophagy does not fully explain ATP13A2 function. In recent years, the functions of ATP13A2 in processes other than autophagy have received more attention.^45^, ^46^, ^47^ Here, our study further refines the effect of ATP13A2 on CRC proliferation and, more importantly, discovers the regulatory role of ATP13A2 in the PPP in CRC for the first time. These results not only complement mechanistic studies of the effects of ATP13A2 on CRC but also further clarify the functions of ATP13A2 beyond autophagy.

As one of the most important pathways promoting tumourigenesis and development, the activation of the PPP has a substantial contribution to tumour proliferation and the ability to resist oxidative stress, as mainly manifested by changes in the product levels of R‐5‐P,^10^, ^38^ NADPH^15^ and ROS.^48^ Unfortunately, despite the well‐known effects of the PPP on tumours, the genes regulating the PPP remain to be further studied. Here, we found that the down‐regulation of ATP13A2 decreased R‐5‐P and NADPH production and increased the level of ROS in CRC. Subsequent experiments further revealed that the effect of ATP13A2 on the PPP manifested as changes in both proliferation and antioxidative stress. Taken together, our results revealed a novel and important PPP regulatory gene, providing a new target for the development of specific PPP inhibitors in the future.

TFEB, which belongs to the MiTF/TFE family, has been identified as a key regulator of lysosomal biogenesis.^49^ TFEB plays a diverse range of roles, including but not limited to the regulation of autophagy, lysosomal biogenesis, and lipid catabolism mediators’ expression. According to recent evidence, TFEB is activated in response to mitochondrial^50^ and ER^51^ stress, suggesting an important role in cellular adaptation to stress. In addition, in response to nutrient deprivation, cancer cells use TFEB to promote their own survival.^52^ Although many functions of TFEB have been identified, the relationship of TFEB to glucose metabolism‐related genes is poorly understood. In our study, we confirmed the transcriptional regulation of PGD by TFEB, revealing the existence of the ATP13A2‐TFEB‐PGD axis. The findings suggest a connection between TFEB and glucose metabolism, which has significant implications for cancer prevention and enhances our understanding of the biological functions of TFEB in cancer.

As the rate‐limiting enzyme of the PPP, PGD plays an extremely important role in the regulation and coordination between the oxidative PPP and glycolysis of tumour cells and subsequent tumour growth.^14^ Although the role of PGD has been documented in various types of tumours, the relationship between PGD and CRC remains unclear. Here, we show for the first time the important role of PGD in CRC development elucidate the specific mechanism of the ATP13A2‐TFEB‐PGD signaling axis, complement the relationship between PGD and tumours, and partially explain the effect of ATP13A2 on CRC. Interestingly, PGD plays an important role in the plasticity and function of regulatory T cells (Tregs).^53^ This result provides support for the relationship between ATP13A2 and immunity, which is worthy of further exploration.

PDO and PDX models have recently emerged as robust preclinical models with the potential to predict clinical outcomes in patients.^54^, ^55^ However, few studies investigating PPP‐related genes have applied these models to further substantiate their findings. Here, we found that ATP13A2 knockdown significantly inhibited CRC growth in PDO and PDX models. Subsequently, using the AOM/DSS mouse model of CRC, we obtained a better understanding of the key role of ATP13A2 in tumour growth and confirmed the clinical therapeutic potential of ATP13A2.Future studies will investigate ATP13A2 as a metabolism‐targeting drug that might be a new treatment strategy for CRC.

## 5 CONCLUSION

This work proved that ATP13A2 overexpression enhances PPP activity and promotes CRC growth. Mechanistically, ATP13A2 retains more TFEB in the nucleus and increases the transcription of PGD by dephosphorylating TFEB, ultimately enhancing PPP activity. Our study identifies a novel gene regulating the PPP and suggests ATP13A2 as a promising potential therapeutic target for CRC.

## CONFLICT OF INTEREST STATEMENT

The authors declare that there is no conflict of interest that could be perceived as prejudicing the impartiality of the research reported.

## Supporting information

Figure S1: The three PPP‐related genes were analyzed using bioinformatics. (A) ATP13A2 mRNA levels in FHC, SW480, SW620, HT29, LOVO and RKO cells were reported in the CCLE database. (B) Cell proliferation was assessed using a CCK‐8 assay. (C) Cancer cell mutation status of ATP13A2 in the COSMIC database. (D) DNA methylation levels of ATP13A2 in colorectal cancer and normal tissues in the TCGA database.

Figure S2: ATP13A2 promotes CRC cell proliferation in vitro and in vivo. (A) Western blot analysis and sequencing data after the successful construction of ATP13A2 knockout cells. (B) Representative image of established stable cell lines showing that the ATP13A2 overexpression vector was constructed and stably expressed in RKO cells. (C) Cell proliferation curve obtained using the CCK‐8 assay. (D) EdU%, which represents the EdU labelling index (%), was calculated as the number of EdU‐positive cells/total number of DAPI‐positive cells. (E) Self‐renewal ability was detected by performing plate colony formation assays. (F and G) Apoptosis rate (%) of each group. (H) Western blot analysis after overexpression of ATP13A2 in ATP13A2 knockout cells. (I) Cell proliferation curve obtained using the CCK‐8 assay. (J) The expression of apoptosis‐associated proteins in ATP13A2 knockout or overexpressing cells. (K) Photograph and quantification of the size of excised subcutaneous tumours (*n* = 5 mice per group). (L) Tumour volume curve and (M) tumour weight graph. (N) Gross appearance of tumours harvested from mice. (O) HE staining and Ki67 immunostaining of transplanted tumour tissues and Ki67 protein immunohistochemical histological score (H‐score). (P and Q) Detection of apoptosis‐related proteins from CRC xenograft samples using Western Blot. (R) Negative control for IHC. All data are presented as means ± SD (*n* = 3 independent experiments). *p ≤ .05; **p ≤ .01; ***p ≤ .001; and ****p ≤ .0001.

Figure S3: ATP13A2 enhances PPP activity in CRC cells. (A) ATP13A2‐KO cells were cultured for 12 h with complete medium (Medium) containing 10 mM ^13^C‐1,2‐D‐glucose. The medium was used to analyze PPP flux with LC‒MS. (B and C) Analysis of lactate and glucose uptake by ATP13A2‐KO cell lines using the colorimetric method. (D) NADP^+^/NADPH levels in ATP13A2‐KO cells. (E) FACS analysis (left panel) and statistical results (right panel) for ROS levels in ATP13A2‐KO cells. (F) Effect of H_2_O_2_ concentrations. CRC cells (5 × 10^3^/well) were seeded in 96‐well culture plates. After an incubation for 24 h to allow cells to attach, the cells were continuously exposed to different concentrations of H_2_O_2_ for 24 h. Cell viability was quantified using a CCK‐8 assay. (G) Calcein‐AM/PI double staining of living cells and dead cells. Living cells were stained with Calcein‐AM (green), and dead cells were stained with PI (red). (H) FACS analysis (left panel) and statistical results (right panel) for ROS levels after treatment with 200 μmol for 24 h. (I) NADP^+^/NADPH levels in ATP13A2‐KO cells after treatment with 200 μmol for 24 h. All data are presented as means ± SD (*n* = 3 independent experiments). *p ≤ .05; **p ≤ .01; ***p ≤ .001; ****p ≤ .0001.

Figure S4: ATP13A2 enhances the PPP by regulating PGD expression. (A) Expression levels of PPP enzymes in ATP13A2‐KO and ATP13A2‐OE cells were tested with qRT‐PCR. (B) Cell proliferation was assessed using a CCK‐8 assay. (C) EdU%, which is the EdU labelling index (%), was calculated as the number of EdU‐positive cells/total number of DAPI‐positive cells. (D) Using [U^13^C]‐labelled glucose, RU‐5‐P and R‐5‐P levels generated from PPP flux were detected with LC‒MS. (E) Calcein‐AM/PI double staining of living cells and dead cells. Living cells were stained with Calcein‐AM (green), and dead cells were stained with PI (red). (F) FACS analysis (left panel) and statistical results (right panel) for ROS levels after treatment with 200 μmol for 24 h. (G) FACS analysis (left panel) and statistical results (right panel) for ROS levels after treatment with 200 μmol for 24 h. All data are presented as means ± SD (*n* = 3 independent experiments). *p ≤ .05; **p ≤ .01; ***p ≤ .001; ****p ≤ .0001.

Figure S5: Validation of the ATP13A2‐TFEB‐PGD axis in collected tissue samples. (A) Detection of nuclear and cytoplasmic proteins in SW480. (B) RKO cells using western blot. (C) The western blot detected the expression of ATP13A2, TFEB and PGD in 12 pairs of cancer and paraneoplastic tissues.

Figure S6: The construction of PDX and AOM/DSS models. (A) Validation of ATP13A2 knockdown efficiency in PDX tissue. (B) Gross appearance of tumours harvested from mice. (C) Expression of PGD in different groups of PDX tissues. (D and E) Validation of ATP13A2 knockout mice. (F) Macroscopic views of the tumour area in the colon after tumour initiation in different groups. (G) Expression of PGD in intestinal tumours of ATP13A2^+/+^ and ATP13A2^−/‐^ mice in the AOM‐DSS group. (H) Negative control for IHC.

Table S1: Information about primer

Table S2: Information about antibodies
