# Supplementary figures and images for: ATP13A2 activates the pentose phosphate pathway to promote colorectal cancer growth though TFEB‐PGD axis
Source: Clin Transl Med. 2023 May 26;13(5):e1272. doi: 10.1002/ctm2.1272 (PMC10220388; doi:10.1002/ctm2.1272)

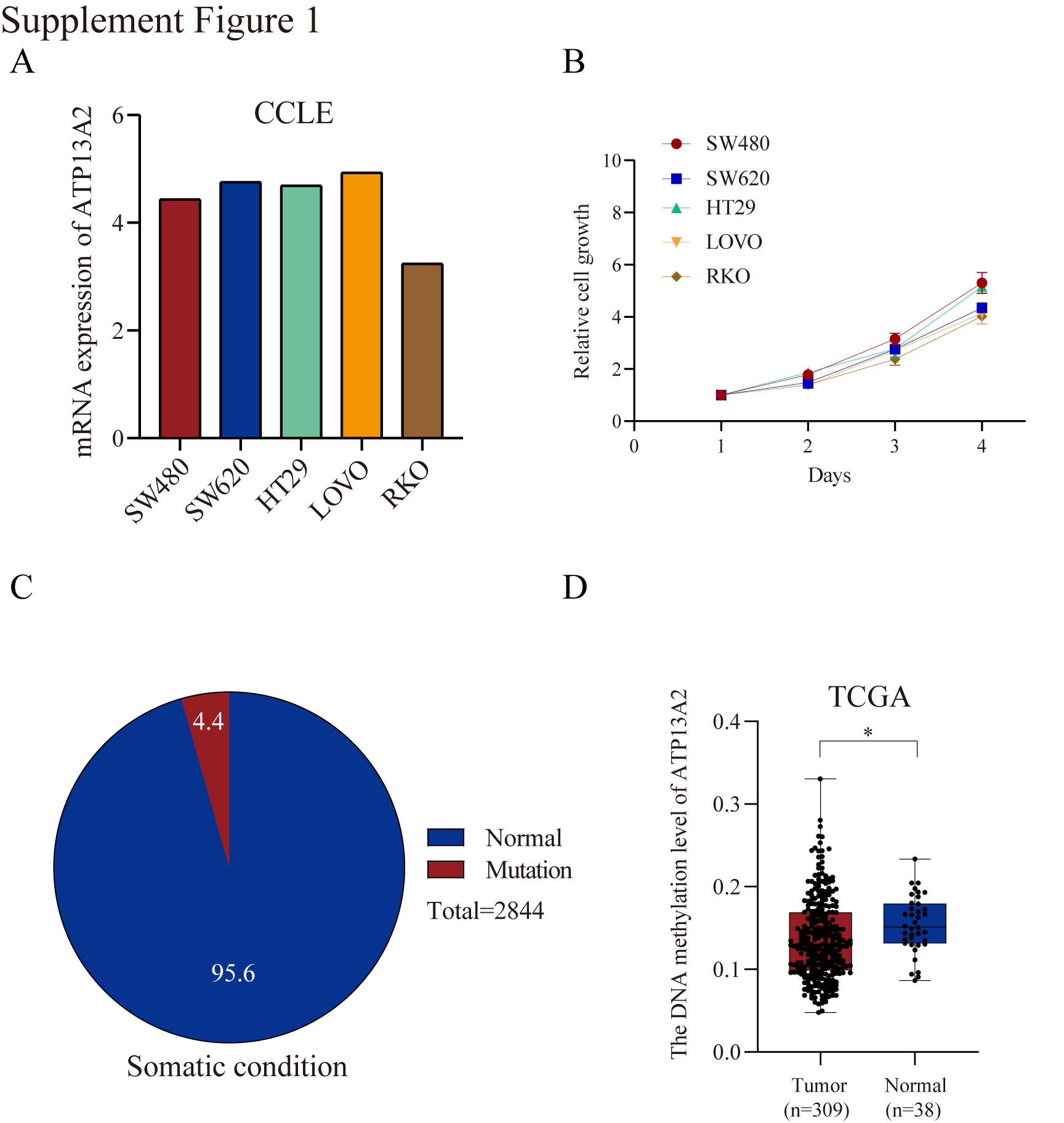

Supplement: Supplementary file 1 — Figure S1: The three PPP‐related genes were analyzed using bioinformatics. (A) ATP13A2 mRNA levels in FHC, SW480, SW620, HT29, LOVO and RKO cells were reported in the CCLE database. (B) Cell proliferation was assessed using a CCK‐8 assay. (C) Cancer cell mutation status of ATP13A2 in the COSMIC database. (D) DNA methylation levels of ATP13A2 in colorectal cancer and normal tissues in the TCGA database. [file CTM2-13-e1272-s007.tif]

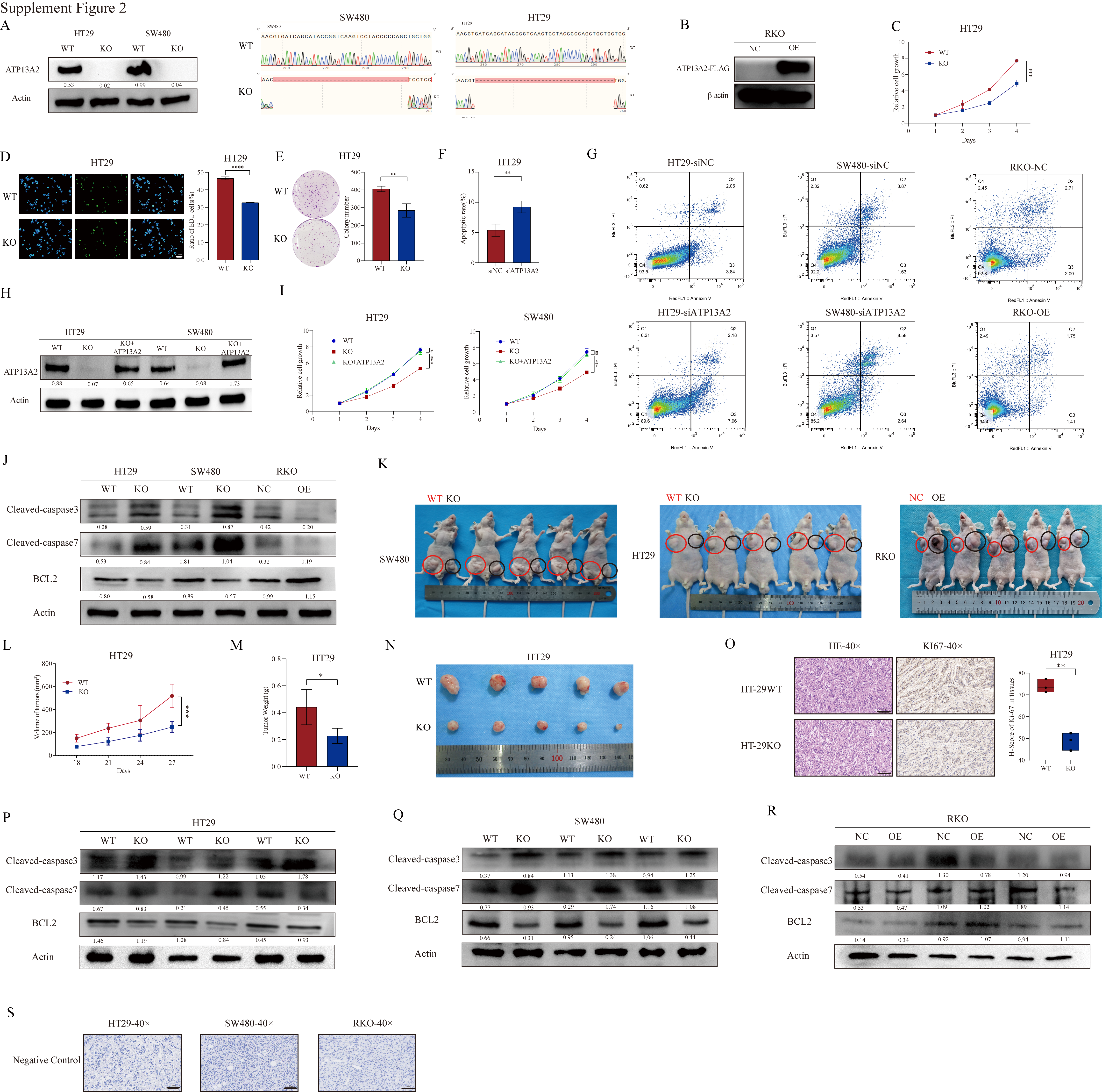

Supplement: Supplementary file 2 — Figure S2: ATP13A2 promotes CRC cell proliferation in vitro and in vivo. (A) Western blot analysis and sequencing data after the successful construction of ATP13A2 knockout cells. (B) Representative image of established stable cell lines showing that the ATP13A2 overexpression vector was constructed and stably expressed in RKO cells. (C) Cell proliferation curve obtained using the CCK‐8 assay. (D) EdU%, which represents the EdU labelling index (%), was calculated as the number of EdU‐positive cells/total number of DAPI‐positive cells. (E) Self‐renewal ability was detected by performing plate colony formation assays. (F and G) Apoptosis rate (%) of each group. (H) Western blot analysis after overexpression of ATP13A2 in ATP13A2 knockout cells. (I) Cell proliferation curve obtained using the CCK‐8 assay. (J) The expression of apoptosis‐associated proteins in ATP13A2 knockout or overexpressing cells. (K) Photograph and quantification of the size of excised subcutaneous tumours (n = 5 mice per group). (L) Tumour volume curve and (M) tumour weight graph. (N) Gross appearance of tumours harvested from mice. (O) HE staining and Ki67 immunostaining of transplanted tumour tissues and Ki67 protein immunohistochemical histological score (H‐score). (P and Q) Detection of apoptosis‐related proteins from CRC xenograft samples using Western Blot. (R) Negative control for IHC. All data are presented as means ± SD (n = 3 independent experiments). *p ≤ .05; **p ≤ .01; ***p ≤ .001; and ****p ≤ .0001. [file CTM2-13-e1272-s003.tif]

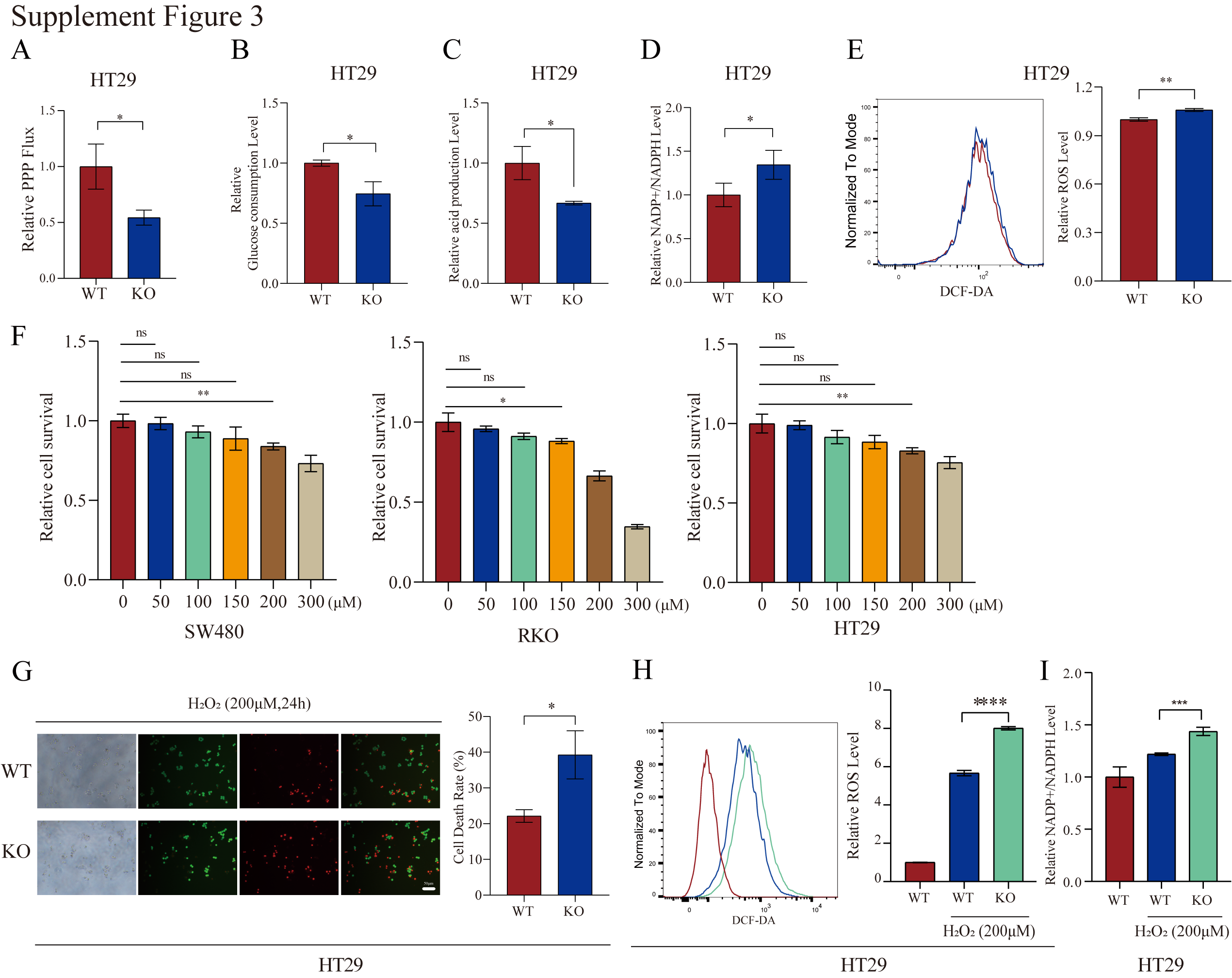

Supplement: Supplementary file 3 — Figure S3: ATP13A2 enhances PPP activity in CRC cells. (A) ATP13A2‐KO cells were cultured for 12 h with complete medium (Medium) containing 10 mM 13C‐1,2‐D‐glucose. The medium was used to analyze PPP flux with LC‒MS. (B and C) Analysis of lactate and glucose uptake by ATP13A2‐KO cell lines using the colorimetric method. (D) NADP+/NADPH levels in ATP13A2‐KO cells. (E) FACS analysis (left panel) and statistical results (right panel) for ROS levels in ATP13A2‐KO cells. (F) Effect of H2O2 concentrations. CRC cells (5 × 10^3^/well) were seeded in 96‐well culture plates. After an incubation for 24 h to allow cells to attach, the cells were continuously exposed to different concentrations of H2O2 for 24 h. Cell viability was quantified using a CCK‐8 assay. (G) Calcein‐AM/PI double staining of living cells and dead cells. Living cells were stained with Calcein‐AM (green), and dead cells were stained with PI (red). (H) FACS analysis (left panel) and statistical results (right panel) for ROS levels after treatment with 200 μmol for 24 h. (I) NADP+/NADPH levels in ATP13A2‐KO cells after treatment with 200 μmol for 24 h. All data are presented as means ± SD (n = 3 independent experiments). *p ≤ .05; **p ≤ .01; ***p ≤ .001; ****p ≤ .0001. [file CTM2-13-e1272-s002.tif]

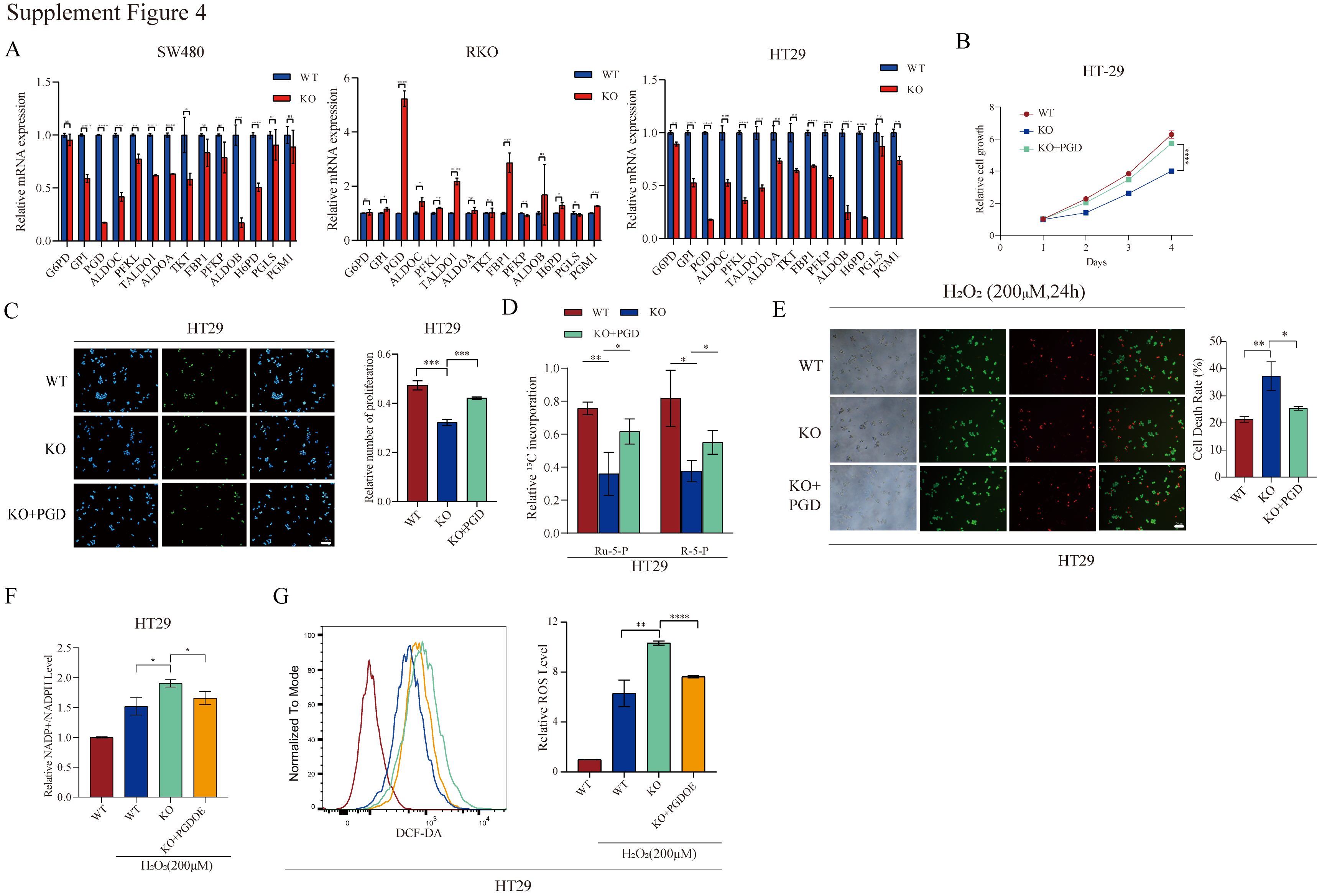

Supplement: Supplementary file 4 — Figure S4: ATP13A2 enhances the PPP by regulating PGD expression. (A) Expression levels of PPP enzymes in ATP13A2‐KO and ATP13A2‐OE cells were tested with qRT‐PCR. (B) Cell proliferation was assessed using a CCK‐8 assay. (C) EdU%, which is the EdU labelling index (%), was calculated as the number of EdU‐positive cells/total number of DAPI‐positive cells. (D) Using [U13C]‐labelled glucose, RU‐5‐P and R‐5‐P levels generated from PPP flux were detected with LC‒MS. (E) Calcein‐AM/PI double staining of living cells and dead cells. Living cells were stained with Calcein‐AM (green), and dead cells were stained with PI (red). (F) FACS analysis (left panel) and statistical results (right panel) for ROS levels after treatment with 200 μmol for 24 h. (G) FACS analysis (left panel) and statistical results (right panel) for ROS levels after treatment with 200 μmol for 24 h. All data are presented as means ± SD (n = 3 independent experiments). *p ≤ .05; **p ≤ .01; ***p ≤ .001; ****p ≤ .0001. [file CTM2-13-e1272-s006.tif]

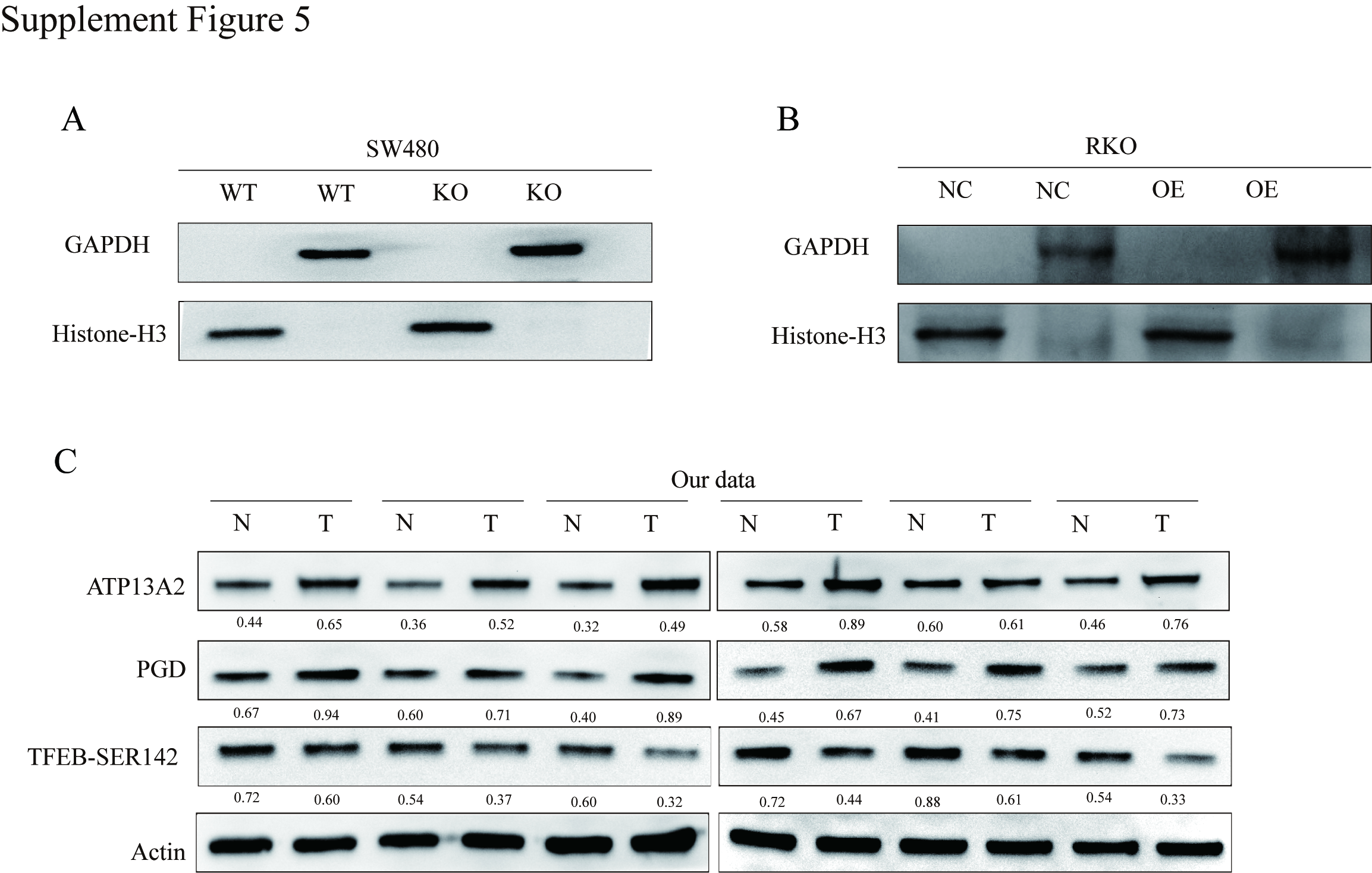

Supplement: Supplementary file 5 — Figure S5: Validation of the ATP13A2‐TFEB‐PGD axis in collected tissue samples. (A) Detection of nuclear and cytoplasmic proteins in SW480. (B) RKO cells using western blot. (C) The western blot detected the expression of ATP13A2, TFEB and PGD in 12 pairs of cancer and paraneoplastic tissues. [file CTM2-13-e1272-s005.tif]

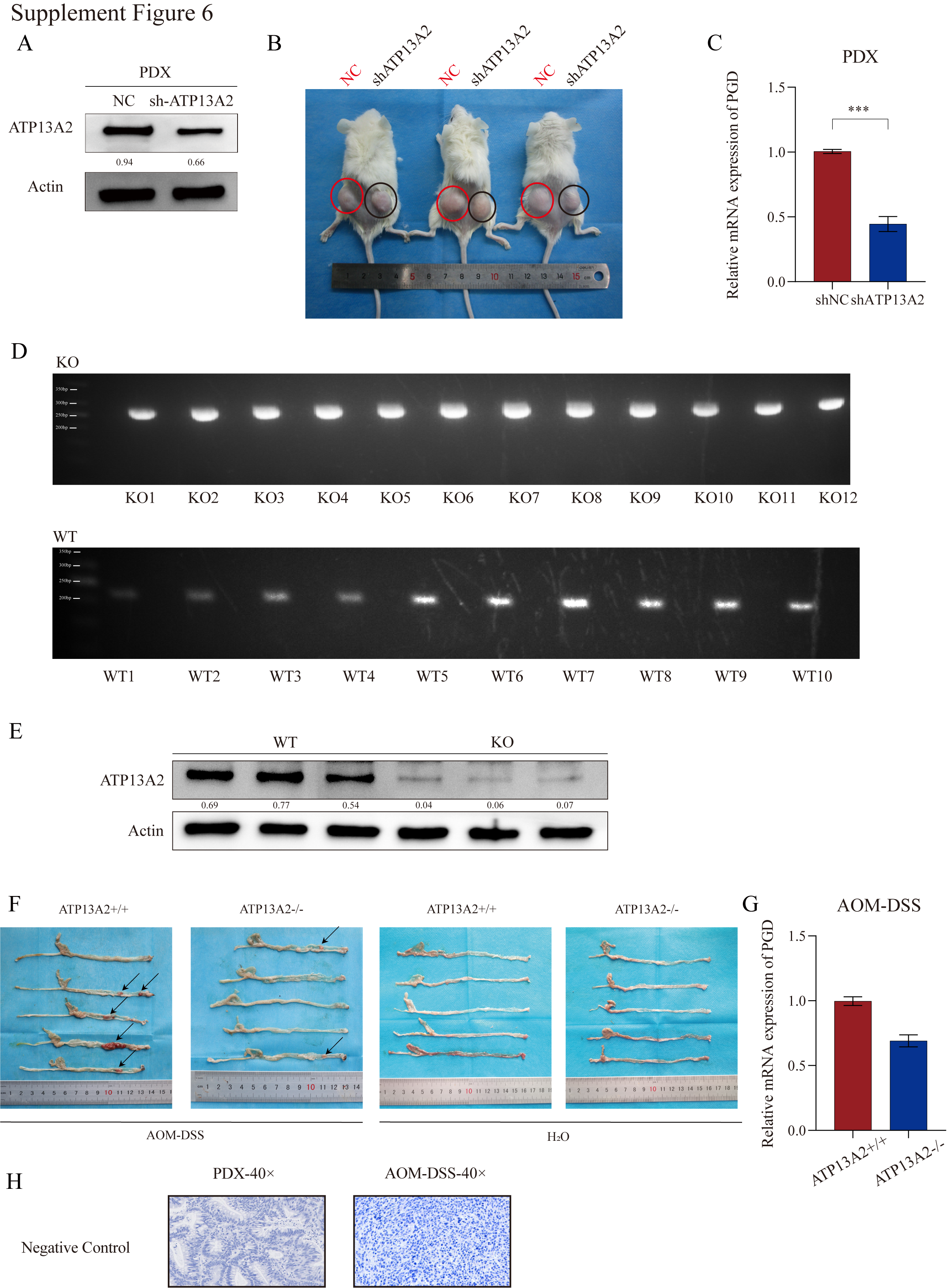

Supplement: Supplementary file 6 — Figure S6: The construction of PDX and AOM/DSS models. (A) Validation of ATP13A2 knockdown efficiency in PDX tissue. (B) Gross appearance of tumours harvested from mice. (C) Expression of PGD in different groups of PDX tissues. (D and E) Validation of ATP13A2 knockout mice. (F) Macroscopic views of the tumour area in the colon after tumour initiation in different groups. (G) Expression of PGD in intestinal tumours of ATP13A2+/+ and ATP13A2−/‐ mice in the AOM‐DSS group. (H) Negative control for IHC. [file CTM2-13-e1272-s008.tif]
